# The Impact of Unpredictability on Dyspnea Perception, Anxiety and Interoceptive Error Processing

**DOI:** 10.3389/fphys.2019.00535

**Published:** 2019-05-03

**Authors:** Yafei Tan, Omer Van den Bergh, Jiang Qiu, Andreas von Leupoldt

**Affiliations:** ^1^Faculty of Psychology, Southwest University, Chongqing, China; ^2^Health Psychology, KU Leuven, Leuven, Belgium

**Keywords:** dyspnea, unpredictability, anxiety, interoception, error-related negativity

## Abstract

Dyspnea is a prevalent interoceptive sensation and the aversive cardinal symptom in many cardiorespiratory diseases as well as in mental disorders. Especially the unpredictability of the occurrence of dyspnea episodes has been suggested to be highly anxiety provoking for affected patients. Moreover, previous studies demonstrated that unpredictable exteroceptive stimuli increased self-reports and electrophysiological responses of anxiety such as the startle probe N100 as well as amplified the processing of errors as reflected by greater error-related negativity (ERN). However, studies directly examining the role of unpredictability on dyspnea perception, anxiety, and error processing are widely absent. Using high-density electroencephalography, the present study investigated whether unpredictable compared to predictable dyspnea would increase the perception of dyspnea, anxiety and interoceptive error processing. Thirty-two healthy participants performed a respiratory forced choice reaction time task to elicit an interoceptive ERN during two conditions: an unpredictable and a predictable resistive load-induced dyspnea condition. Predictability was manipulated by pairing (predictable condition) or not pairing (unpredictable condition) dyspnea with a startle tone probe. Self-reports of dyspnea and affective state as well as the startle probe N100 and interoceptive ERN were measured. The results demonstrated greater dyspnea unpleasantness in the unpredictable compared to the predictable condition. *Post hoc* analyses revealed that this was paralleled by greater anxiety, and greater amplitudes for the startle probe N100 and the interoceptive ERN during the unpredictable relative to the predictable condition, but only when the unpredictable condition was experienced in the first experimental block. Furthermore, higher trait-like anxiety sensitivity was associated with higher ratings for dyspnea unpleasantness and experimental state anxiety ratings. The present findings suggest that unpredictability increases the perception of dyspnea unpleasantness. This effect seems related to increased state and trait anxiety and interoceptive error processing, especially when upcoming dyspnea is particularly unpredictable, such as in early experimental phases. Future studies are required to further substantiate these findings in patients suffering from dyspnea.

## Introduction

Dyspnea is the subjective experience of uncomfortable breathing (American Thoracic Society, 1999) and a prevalent interoceptive sensation. It is an impairing symptom in various cardiopulmonary diseases such as asthma and chronic obstructive pulmonary disease (COPD) as well as in mental disorders such as anxiety disorders (Smoller et al., 1996; Solano et al., 2006) and associated with significant reductions in functioning and quality of life (American Thoracic Society, 1999; Parshall et al., 2012; Laviolette and Laveneziana, 2014). Dyspnea greatly increases individual and socioeconomic burden and is a main cause of morbidity and mortality worldwide (Booth et al., 2003; Janssen et al., 2015; Mentz et al., 2015; Currow et al., 2018; Hutchinson et al., 2018).

Recent research has suggested that the perception of dyspnea is a complex individual interpretation process of respiratory input that is modulated by affective and cognitive factors (Lansing et al., 2009; Janssens et al., 2011; Hayen et al., 2013; Herigstad et al., 2017; Spathis et al., 2017; Van den Bergh et al., 2017; von Leupoldt, 2017; Similowski, 2018). For example, previous studies have demonstrated that high levels of state and trait anxiety are associated with elevated reports of dyspnea in everyday-life settings (Xu et al., 2008) as well as in experimental studies in healthy individuals (Alius et al., 2013; Stoeckel et al., 2015; Sharma et al., 2016; Herzog et al., 2018) and patients with cardiopulmonary diseases (Livermore et al., 2008; Reijnders et al., 2019; von Leupoldt et al., 2011). Dyspnea can be classified into predictable and unpredictable dyspnea episodes (Simon et al., 2014). Especially unpredictable dyspnea episodes have been suggested to create a strong sense of loss of control, to be specifically anxiety provoking and to amplify the perception of dyspnea (Booth et al., 2018; Lovell et al., 2018). For example, in a qualitative study patients with COPD and lung cancer reported that unpredictable dyspnea episodes were stronger and more unpleasant compared to predictable dyspnea episodes (Linde et al., 2018). However, experimental studies directly examining the role of unpredictability on the perception of dyspnea and its’ relationships with individual state and trait anxiety levels are widely absent.

In contrast, previous studies using other exteroceptive sensory stimuli have demonstrated that unpredictability is indeed related to increased anxiety and hypervigilance (Grillon et al., 2004, 2008; Schmitz et al., 2011; Shankman et al., 2013; Jackson et al., 2015; Wieser et al., 2016; Speed et al., 2017). For example, unpredictable compared to predictable presentations of electric shocks and/or visual stimuli led to heightened subjective anxiety ratings and increased amplitudes of the startle N100 event-related potential as an electrophysiological marker for increased anxious hyper-vigilance in healthy individuals (Nelson et al., 2015a; Nelson and Hajcak, 2017). This was paralleled by studies showing not only increased anxiety reports but also increased electrophysiological amplitudes of the error-related negativity (ERN) for errors committed in a forced choice Flanker task during unpredictable relative to predictable acoustic stimulation (Jackson et al., 2015; Speed et al., 2017). It has been suggested that errors are, similar to external threats, motivationally salient and threatening endogenous events (Hajcak and Foti, 2008; Hajcak, 2012). Accordingly, increased ERNs have been proposed as an electrophysiological marker for elevated threat sensitivity (Hajcak, 2012). Given that unpredictability has been shown to relate to increased anxiety and electrophysiological markers of anxiety (startle N100, ERN), it can be speculated that it would also increase the perception of dyspnea, especially as higher anxiety is known to be related to higher dyspnea.

Notably, experimental studies directly examining the impact of unpredictability of dyspnea episodes on the perception of dyspnea and its’ relationships with individual anxiety levels as well as on electrophysiological markers of anxiety (startle N100, ERN) are absent. However, an improved understanding of the interactions between the unpredictability of dyspnea and anxiety may identify potential targets for non-pharmacological interventions for dyspnea in patients with cardiopulmonary and mental diseases.

Therefore, using high-density EEG, the present study examined the effects of unpredictability on the perception of dyspnea, anxiety, and error processing in healthy individuals. Specifically, healthy participants underwent short states of resistive load induced dyspnea either in a predictable or in an unpredictable manner by combining the loads with startle tone probes or not, respectively. Load magnitudes were similar in both conditions and the N100 in response to the tone probes was measured. Simultaneously, error processing was examined using the interoceptive error-related negativity (intERN) in an interoceptive forced choice reaction time task (Tan et al., 2018). Here, individuals received inspiratory occlusions of two different durations and indicated whether each occlusion was short or long, respectively. We hypothesized that compared to the predictable condition, the unpredictable condition would result in increased reports of dyspnea and anxiety as well as increased hyper-vigilance as reflected by increased amplitudes of the tone probe N100. Moreover, we expected greater intERN amplitudes as well as higher levels of performance accuracy. In additional explorative analyses, we examined whether these effects would be most pronounced in individuals with high levels of interoception-related negative affect.

## Materials and Methods

### Participants

Thirty-two healthy participants (22 females) with normal vision and hearing were tested after providing written informed consent in accordance with the Declaration of Helsinki. All participants reported the absence of cardiovascular, respiratory, neurological, psychiatric, or psychological diseases. Other exclusion criteria included respiratory symptoms within the preceding 2 weeks, alcohol or drug intoxication, nicotine consumption, insufficient pulmonary function, pregnancy, and not being a Dutch speaker. Sufficient lung function (FEV_1_ in % predicted >80%) was confirmed by a standard spirometry test (Miller et al., 2005). Before the main task, all participants completed a battery of questionnaires including the validated Anxiety Sensitivity Index (ASI), which is a 16-item self-report questionnaire assessing fear of anxiety-related physical sensations (Reiss et al., 1986). The items (e.g., “When my chest feels tight, I worry I could choke to death.”) are answered on a five-point scale ranging from 0 (*very little*) to 4 (*very much*) with greater summary scores reflecting higher anxiety sensitivity. Participants received either one course credit or 8 Euro per hour for their participation. The study was approved by the Social and Societal Ethics Committee of the University of Leuven (G-2018-02-1123). Participant characteristics are presented in Table 1.

**Table 1:** Mean (SD) characteristics of participants.

Characteristics	Data
Gender (female/male, No.)	(22/10)
Age (years)	25.19 (6.47)
FEV_1_ (L)	4.08 (0.93)
FEV_1_ (% predicted)	98.38 (7.25)
Anxiety sensitivity index score	24.97 (8.47)
Breathing frequency	19.45 (5.53)

*FEV_1_ = forced expiratory volume in 1 s.*

### Experimental Set Up

The experiment included a respiratory forced choice reaction time task using inspiratory occlusions (Tan et al., 2018) with the additional occurrence of brief episodes of predictable or unpredictable dyspnea as induced by inspiratory resistive loads. During the task, participants were seated in a recliner in a sound-attenuated test room, which was connected to an adjacent control room. They were breathing normally via a mouthpiece through a breathing circuit with the nose occluded by a clip. The breathing circuit contained a two-way non-rebreathing valve (Hans Rudolph Inc., Shawnee, OK, United States). The expiratory port of the valve was left free in order to minimize CO_2_ rebreathing and associated hypercapnia. The inspiratory port was connected via reinforced tubing to a pneumotachograph (Hans Rudolph Inc., Shawnee, OK, United States), an occlusion device (Aspire Products, Gainesville, FL, United States) and a loading manifold (Hans Rudolph Inc., Shawnee, OK, United States). Signals of airflow and mouth pressure were sent to a flow-pressure amplifier (series 1110, Hans Rudolph Inc., Shawnee, OK, United States) connected to a data acquisition card (NI PCI-6221 series, National Instruments, Austin, TX, United States), enabling the experimenter to continuously monitor the airflow and mouth pressure on a PC screen. Based on these individual airflow or mouth pressure signals, the occlusion device was used to trigger occlusions manually by the experimenter after the onset of an inspiration. The loading manifold was used to apply inspiratory resistive loads (Hans Rudolph Inc., Shawnee, OK, United States), which induced dyspnea. The questionnaires and the experimental task with instructions, fixation crosses and rating scales were presented on a monitor using Affect software, version 5.0 (Spruyt et al., 2009). Participants’ responses were provided by button press on a PC mouse.

Startle tone probes were presented at 95 dB via a loudspeaker positioned approximately 50 cm behind the participant.

The EEG sensor cap was attached before the task. During each experimental block, 129-channel EEG (Philips Electrical Geodesics Inc., Eugene, OR, United States) was continuously recorded from the scalp with a sampling rate of 250 Hz. Impedance was kept below 50 kΩ using the vertex sensor as reference.

### Experimental Protocol

Similar to our previous study (Tan et al., 2018), a respiratory forced choice reaction time task using inspiratory occlusions was used to study interoceptive error processing. Here, individuals received inspiratory occlusions of two different durations (160 ms vs. 240 ms) every two to six breaths. After each occlusion, the participants indicated as correct and as fast as possible whether the occlusion was short or long, respectively. Responses had to be provided by button press within 1200 ms with a parallel marker signal being sent to the EEG recorder. Errors in this task elicit the interoceptive ERN, which is an event-related potential in the EEG characterized by a negative-going deflection at fronto-central scalp positions within the first 100 ms after error commission (Falkenstein et al., 1991; Gehring et al., 1993).

**FIGURE 1:**
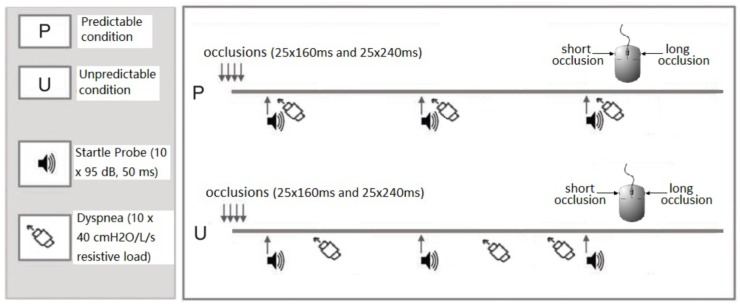
Graphical visualization of the experimental design. In both predictable and unpredictable conditions, the participants performed the respiratory forced choice reaction time task during which they received 50 inspiratory occlusions of two different durations. They indicated via mouse press whether each occlusion was short (160 ms) or long (240 ms), respectively, directly after each occlusion. In the predictable condition, tones were presented every minute and immediately followed by 2 loaded inspirations during which participants inspired through a resistive load (40 cmH2O/L/s). In the unpredictable condition, tones were presented every minute, but the 2 loaded inspirations were presented randomly and not related to the tones. The conditions were announced on the screen right before the blocks started and an additional specific color frame remained on the screen for the whole duration of the block to remind the participants of the condition. The order of the predictable and unpredictable conditions was counterbalanced across participants.

The participants performed the task under two experimental conditions (see Figure 1): a predictable dyspnea condition (P) and an unpredictable dyspnea condition (U). Each condition included two blocks during which 50 occlusions (25 short and 25 long occlusions in random order), 10 startle tone probes (95 dB, 50 ms duration) and 10 resistive loads (40 cmH_2_O/l/s) were presented. During the predictable dyspnea condition (P), one tone probe was presented every minute and immediately followed by resistive load-induced dyspnea for 2 inspirations. During the unpredictable dyspnea condition (U), one tone probe was presented every minute, but the resistive load-induced dyspnea was presented randomly for 2 inspirations and was not related to the tones. The conditions were announced on the screen right before the blocks started with an additional specific color frame being presented on the screen for the whole duration of each block. The color and order of the conditions were counterbalanced such that half of the participants underwent the task in the order PUPU and the other half in the order UPUP.

Each block lasted approximately 12 min and was followed by rating scales and a resting period. In order to familiarize the participants with the short and long occlusions, conditions and ratings, detailed instructions, and practice trials were presented prior to the actual experiment. The practice trials included three parts: in the first part, 4 short occlusions and 4 long occlusions were presented to the participants and they were informed about the duration of each occlusion (i.e., short vs. long). Then, 4 short and 4 long occlusion trials were performed without information about the duration and responses had to be given. In the next practice trial, 4 short and 4 long occlusion trials and 2 startle tone probes paired with 2 subsequent predictable resistive load presentations (mimicking the predictable P condition) were presented and responses as well as ratings (see below) had to be given. In the last practice trial, 4 short and 4 long occlusion trials as well as 2 startle tone probes with 2 unpaired (unpredictable) resistive loads (mimicking the unpredictable U condition) were presented and responses as well as ratings had to be given.

### Ratings

At the end of each block, the participants provided ratings of dyspnea intensity (‘How intense did your breathlessness feel?’) and dyspnea unpleasantness (‘How unpleasant did your breathlessness feel?’). Ratings were provided on a visual analog scale (Aitken, 1969) ranging from 0 (‘not noticeable/not unpleasant’) to 100 (‘maximally imaginable intensity/unpleasantness’), which is a validated instrument to measure experimentally induced dyspnea (Parshall et al., 2012). In addition, ratings of affective valence (‘How unpleasant/pleasant did you feel during the previous block?’) (1 = very unpleasant to 6 = very pleasant), arousal (‘How aroused did you feel during the previous block?’) (1 = not at all aroused to 6 = very aroused) and anxiety (‘How anxious did you feel during the previous block?’) (1 = not at all anxious to 6 = very anxious) were obtained after each block on a 6-point rating scale, respectively. All ratings were provided after each block in order to avoid potential interference with the performance in the continuous respiratory forced choice reaction time task.

### Data Processing and Analysis

First, incomplete respiratory occlusions were removed after visual inspection of the respiratory data. Next, individual averages for error rate, response time and ratings of dyspnea intensity and unpleasantness, valence, arousal and anxiety were calculated across the two blocks for each condition. These behavioral data were compared between the two conditions using paired *t*-tests.

EEG data were processed offline using Brain Electrical Source Analysis Research 6.0 (BESA GmbH, Gräfelfing, Germany). Data were band-pass filtered (0.1 to 30 Hz with an additional notch filter of 50 Hz) and corrected for ocular artifacts(Ille et al., 2002). A spherical spline procedure was employed to interpolate noisy electrodes (Keil et al., 2014). Furthermore, a semiautomatic procedure was used to detect and reject artifacts using the following thresholds: maximum amplitude >200 μV, variance of gradient 0.01 μV/∂, and a maximal gradient of 75 μV/∂T (Tan et al., 2018). Data were then re-referenced to the average reference.

For analyzing the startle probe N100, tone probe-locked epochs with a duration of 1200 ms, including a 200 ms prestimulus and 1000 ms post-stimulus interval, were extracted and averaged across the two blocks for each condition. The 200 ms prestimulus interval was used as the baseline. Similar to previous studies (Nelson et al., 2015a; Nelson and Hajcak, 2017), the startle probe N100 was quantified as the average amplitude at electrodes around FCz (Geodesic net electrodes 6 and Cz) between 90 and 130 ms after stimulus onset. The average number of included probe trials was 7 out of 10 in each block.

For analyzing the interoceptive ERN, response-locked epochs of 1500 ms, including a 500 ms pre-response and 1000 ms post-response interval, were extracted and averaged across the two blocks for each condition. The 500 – 300 ms pre-response interval was used as the baseline (Tan et al., 2018). Trials with response times below 160 ms or above 1200 ms were excluded from averaging. The average number of included error trials and correct trials in each block were 11 out of 50 and 21 out of 50, respectively. To isolate the error effect, difference wave analysis (error minus correct) was applied to determine the interoceptive ERN (Horan et al., 2012; Luck, 2014). The interoceptive ERN (ΔERN) was quantified as the difference of mean amplitudes between 0 and 100 ms after error and correct responses at electrodes around FCz (Geodesic net electrodes 5, 6, and 12).

The mean amplitudes of the startle probe N100 and the interoceptive ERN (ΔERN) were compared between both conditions using paired *t*-tests, respectively. Subsequent inspection of the data revealed significant order effects for some ratings and the EEG data. Therefore, *post hoc* explorative analyses were conducted analyzing task performance, ratings, startle probe N100 and ΔERN using an Order (UPUP vs. PUPU) × Condition (Predictable dyspnea vs. Unpredictable dyspnea) repeated measures analysis of variance (ANOVA).

Finally, explorative correlational analyses (Pearson’s r) were used to investigate potential associations of anxiety sensitivity scores with ratings of dyspnea unpleasantness, anxiety, startle probe N100, and the interoceptive ERN (ΔERN) for both conditions. Statistical analyses were performed with SPSS 24 (IBM Corp., Armonk, United States) using *p* < 0.05 as the level of significance. For correlational analyses, a Bonferroni corrected significance level of *p* < 0.006 (0.05 / 8 tested correlations) was used in order to reduce type 1 error inflation.

## Results

### Behavioral Data

#### Task Performance

Using paired *t*-test, we found no significant difference in error rates between conditions [*t* (31) = 0.23, *p* = 0.82, *d* = 0.03; Table 2]. *Post hoc* ANOVAs demonstrated no additional Order × Condition interaction [*F* (1, 30) = 0.01, *p* = 0.93, η_p_^2^ < 0.01]. Response time in error trials was significantly slower during the unpredictable condition compared to the predictable condition [*t* (31) = 2.86, *p* < 0.01, *d* = 0.23; Table 2], while no difference was observed for correct trials [*t* (31) = 1.81, *p* = 0.08, *d* = 0.10]. *Post hoc* ANOVAs demonstrated no Order × Condition interaction [*F* (1, 30) = 0.34, *p* = 0.57, η_p_^2^ = 0.01] in error trials but an Order × Condition interaction [*F* (1, 30) = 4.66, *p* < 0.05, η_p_^2^ = 0.13] in correct trials.

**Table 2:** Mean (SD) of response time and error rate for both experimental conditions.

	Predictable condition	Unpredictable condition
Response time correct (ms)	713.95 (126.99)	734.26 (127.29)
Response time error (ms)	705.75 (148.35)	737.87 (129.87)
Error rate (%)	35 (14)	35 (13)

#### Ratings

**FIGURE 2:**
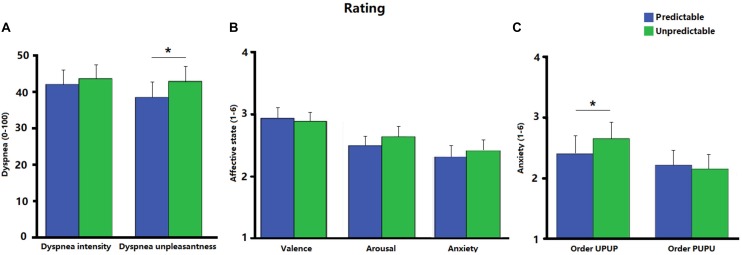
**(A)** Ratings of dyspnea intensity and dyspnea unpleasantness for the predictable and unpredictable condition on a visual analog scale (0–100); **(B)** ratings of affective state including valence, arousal and anxiety for the predictable and unpredictable condition on a scale ranging from 1 to 6; **(C)** ratings of anxiety separately for the condition orders unpredictable- predictable-unpredictable-predictable (UPUP) and predictable-unpredictable-predictable-unpredictable (PUPU) on a scale ranging from 1 to 6. Error bars represent standard errors. ^∗^*p* < 0.05.

Figure 2 shows the ratings of dyspnea intensity, dyspnea unpleasantness, valence, arousal and anxiety for each condition. Ratings for dyspnea unpleasantness were significantly higher during the unpredictable compared to the predictable condition [*t* (31) = 2.23, *p* < 0.05, *d* = 0.18; Figure 2A]. No significant difference was observed between the two conditions for ratings of dyspnea intensity [*t* (31) = 0.91, *p* = 0.37, *d* = 0.07; Figure 2A], valence [*t* (31) = −0.57, *p* = 0.57, *d* = −0.05], arousal [*t* (31) = 1.47, *p* = 0.15, *d* = 0.15], or anxiety [*t* (31) = 1.18, *p* = 0.25, *d* = 0.90; Figure 2B]. *Post hoc* ANOVAs revealed a significant Order × Condition interaction for ratings on anxiety [*F* (1, 30) = 4.31, *p* < 0.05, η_p_^2^ = 0.13]. Follow-up analysis indicated that anxiety was as expected enhanced during the unpredictable compared to the predictable condition when participants underwent the experimental conditions in the order UPUP [*t* (15) = 1.94, *p* < 0.05 (one tailed), *d* = 0.22; Figure 2C], but not in the order PUPU [*t* (15) = −0.81, *p* = 0.22 (one tailed), *d* = −0.07; Figure 2C]. No further significant Order × Condition interactions were observed for ratings of dyspnea unpleasantness [*F* (1, 30) = 2.32, *p* = 0.14, η_p_^2^ = 0.07] and dyspnea intensity [*F* (1, 30) = 1.89, *p* = 0.18, η_p_^2^ = 0.06].

### Electrophysiological Data

#### Startle Probe N100

**FIGURE 3:**
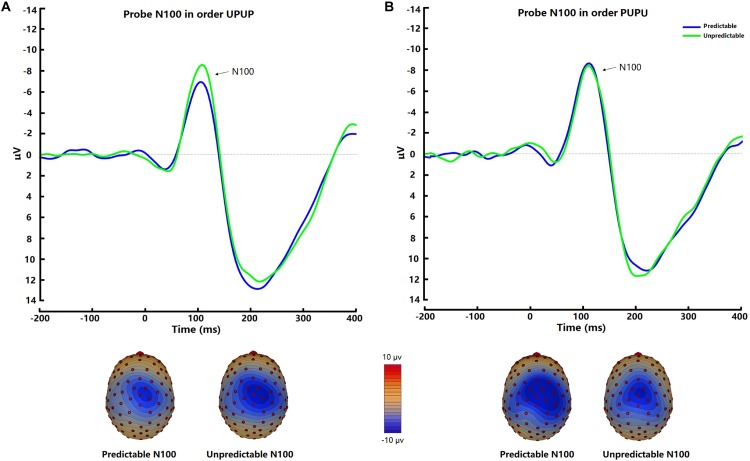
Average waveforms and respective scalp topography plots around FCz for the startle probe N100 for the predictable and unpredictable condition for the **(A)** condition order unpredictable- predictable-unpredictable-predictable (UPUP) and **(B)** condition order predictable-unpredictable-predictable- unpredictable (PUPU).

The startle probe N100 was observed at fronto-central scalp positions and was maximal approximately 110 ms after tone probe onset (Figure 3). There was no significant difference of N100 amplitudes between the two conditions [*t* (31) = −1.00, *p* = 0.32, *d* = −0.11]. *Post hoc* ANOVA analysis indicated a trend for an Order × Condition interaction [*F* (1, 30) = 2.44, *p* = 0.13, η_p_^2^ = 0.08], which was significant after removal of 2 outliers with amplitudes exceeding 3 × SD of the mean [*F* (1, 28) = 5.19, *p* < 0.05, η_p_^2^ = 0.16]. Follow-up analysis revealed higher N100 amplitudes in the unpredictable condition relative to the predictable condition when participants underwent the experimental conditions in the order UPUP [*t* (15) = −2.69, *p* < 0.05, *d* = −0.36; Figure 3A], but not in the order PUPU [*t* (15) = 0.31, *p* = 0.77, *d* = 0.05; Figure 3B].

#### Interoceptive ERN

**FIGURE 4:**
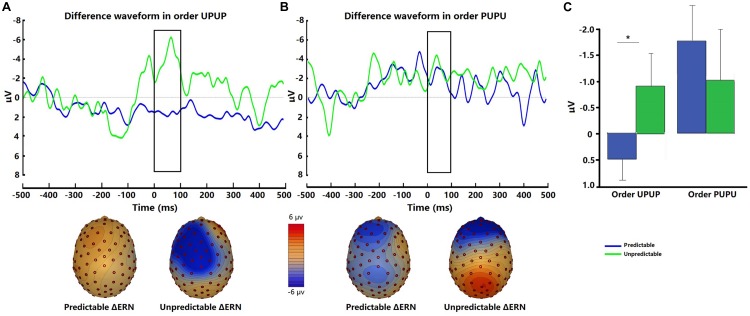
Representative difference waveforms for the interoceptive ERN (error response minus correct response) and respective scalp topography plots around FCz for the predictable and unpredictable condition for **(A)** participant undergoing the condition order unpredictable- predictable-unpredictable-predictable (UPUP) and **(B)** participant undergoing the condition order predictable-unpredictable-predictable-unpredictable (PUPU); **(C)** means of difference waveforms for the interoceptive ERN around FCz across all participants undergoing the condition order UPUP and all participants undergoing condition order PUPU. Error bars represent standard errors. ^∗^*p* < 0.05.

The interoceptive ERN was evident at fronto-central sites when the participants committed errors in the respiratory forced choice reaction time task using occlusions (Figure 4). There was no significant difference in ΔERN amplitudes between the two conditions [*t* (31) = −0.62, *p* = 0.54, *d* = −0.14]. *Post hoc* ANOVA analysis demonstrated a significant Order × Condition interaction [*F* (1, 30) = 4.81, *p* < 0.05, η_p_^2^ = 0.14]. Follow-up tests indicated that ΔERN amplitudes were greater during the unpredictable compared to the predictable condition when participants underwent the experimental conditions in the order UPUP [*t* (15) = −2.62, *p* < 0.05, *d* = −0.83; Figure 4A], but not in the order PUPU [*t* (15) = 0.91, *p* = 0.38, *d* = 0.28; Figure 4B].

**FIGURE 5:**
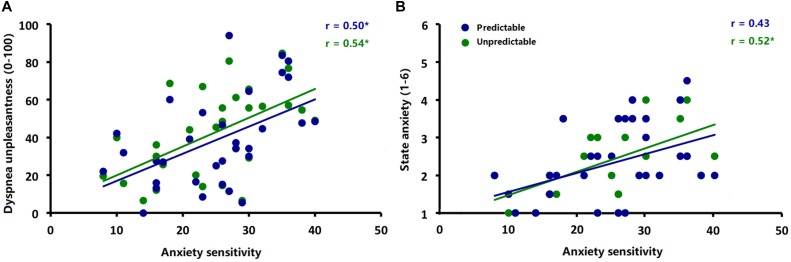
Correlations between **(A)** anxiety sensitivity (ASI scores) and ratings of dyspnea unpleasantness during the predictable and unpredictable conditions and **(B)** anxiety sensitivity (ASI scores) and ratings of anxiety during the predictable and unpredictable conditions. ^∗^*p* < 0.006.

Explorative correlational analysis demonstrated that higher ASI scores were significantly correlated with higher ratings for dyspnea unpleasantness (Figure 5A) in both the predictable [*r* (30) = 0.50, *p* < 0.006] and unpredictable conditions [*r* (30) = 0.54, *p* < 0.006] as well as with higher anxiety ratings (Figure 5B) in the unpredictable condition [*r* (30) = 0.52, *p* < 0.006] but not in the predictable condition [*r* (30) = 0.43, *p* = 0.02]. No significant correlations between ASI scores and amplitudes of startle probe N100 and interoceptive ERN (ΔERN) were observed for either the predictable condition [ERN: *r* (30) = −0.09, *p* = 0.61; N100: *r* (30) = −0.25, *p* = 0.17] or unpredictable condition [ERN: *r* (30) = 0.13, *p* = 0.48; N100: *r* (30) = −0.21, *p* = 0.26].

## Discussion

The present study examined the impact of unpredictability on the perception of dyspnea, anxiety and interoceptive error processing in healthy individuals. Compared to the predictable condition, greater dyspnea unpleasantness was reported in the unpredictable condition. Moreover, state anxiety and amplitudes for the startle probe N100 as well as the interoceptive ERN were higher during the unpredictable relative to the predictable condition, but only when the unpredictable condition was experienced in the first experimental block. In addition, higher trait-like anxiety sensitivity was associated with higher ratings for dyspnea unpleasantness and experimental state anxiety. Taken together, these findings suggest that unpredictability increases the perception of dyspnea, especially its affective unpleasantness dimension. This effect seems related to increased levels of anxiety and electrophysiological measures of anxiety and interoceptive error processing, especially when unpredictability is present in early experimental phases.

The current results are in line with previous findings from qualitative studies which demonstrated that the unpredictability of dyspnea is particularly frightening for patients with cardiopulmonary diseases or cancer and amplifies their perception of dyspnea (Booth et al., 2018; Lovell et al., 2018; Linde et al., 2018). These findings also converge with previous notions that the perception of dyspnea is a subjective interpretation process of respiratory input that is strongly modulated by multiple factors (von Leupoldt and Dahme, 2007; Lansing et al., 2009; Janssens et al., 2011; Hayen et al., 2013; Herigstad et al., 2017; Spathis et al., 2017; Van den Bergh et al., 2017; von Leupoldt, 2017; Similowski, 2018). For example, one model describes that the perception of dyspnea consists of a sensory component (intensity) and an affective component (unpleasantness) (Wilson and Jones, 1991; Meek et al., 2003; von Leupoldt and Dahme, 2005; Lansing et al., 2009). Research has demonstrated that these two components can vary independently and that the affective component of dyspnea perception might be particularly vulnerable to emotional and cognitive influences (von Leupoldt et al., 2006, 2007, 2008, 2017; Banzett et al., 2008; Wan et al., 2009, 2012; Carrieri-Kohlman et al., 2010). The current results further extend these findings by demonstrating that unpredictability increased the unpleasantness, but not the intensity of perceived dyspnea. However, the precise mechanism underlying this effect remains unclear from the current study, but presumably involves elevated anxiety induced by unpredictability.

In line with this notion, the present *post hoc* analyses revealed not only greater self-reports of anxiety, but also higher amplitudes for the startle probe N100 during the unpredictable relative to the predictable condition, which can be interpreted as increased anxious hyper-vigilance during unpredictable dyspnea. These findings support previous experimental observations in healthy individuals, which demonstrated increased ratings of anxiety and distress in unpredictable relative to predictable dyspneic conditions (Acheson et al., 2007; Schroijen et al., 2016). This was accompanied by increased physiological responses of anxiety such as increased electrodermal and/or startle eye blink responses (Pappens et al., 2012; Schroijen et al., 2016; Benke et al., 2018), especially in individuals with high dyspnea-specific fear (Benke et al., 2018).

Moreover, the present results are consistent with a large body of literature demonstrating that unpredictability increases self-reports and (electro)physiological responses of anxiety when using exteroceptive stimuli (Grillon et al., 2004, 2006; Nelson and Shankman, 2011; Schmitz et al., 2011; Nelson et al., 2015a; Nelson and Hajcak, 2017). For example, Nelson et al. (2015a) showed startle probe N100 enhancement during the unpredictable threat-of-shock condition, but not during the predictable condition or during a safe control condition. Similarly, Nelson and Hajcak (2017) found that unpredictability increased the amplitude of the startle probe N100 in anticipation of both aversive shocks and unpleasant pictures.

In addition, unpredictable dyspnea episodes also had an effect on the neural response to errors committed in the interoceptive forced choice reaction time task. Similar to the study of Jackson et al. (2015), in which unpredictable relative to predictable tones increased the amplitudes of the exteroceptie ERN in a Flanker task, the current study found enhanced amplitudes of the interoceptive ERN during the unpredictable relative to the predictable dyspnea condition. These data suggest that unpredictability potentiates a neural bias toward errors which can be interpreted as a type of endogenous threat (Hajcak and Foti, 2008; Hajcak, 2012). In the studies by Jackson et al. (2015) and Speed et al. (2017), the authors further reported improved task accuracy reflecting increased task vigilance during unpredictable relative to predictable contexts. However, the present study observed no significant difference of error rates between the two conditions, but merely a slowing in reaction times in the unpredictable condition without any correlations between error rates and reaction times with ratings of dyspnea. One possible reason is that, in the present study, the task was more difficult than the Flanker task used by the above mentioned studies (Tan et al., 2018). Alternatively, these two studies manipulated the (un)predictability by rather neutral tone sequences, whereas the present study used more aversive dyspnea stimuli, which potentially impair performance and the ability to function to a greater extent (Laveneziana, 2010; Kessler et al., 2011; Johnson et al., 2014). This interpretation is in line with a set of previous studies suggesting that experimentally induced dyspnea as well as its anxious anticipation negatively impact on several domains of functioning such as face recognition (Vinckier et al., 2018), cognition in a mobility task (Nierat et al., 2016), response inhibition (Sucec et al., 2018b) as well as the neural processing of affective pictures (Juravle et al., 2014; Juravle et al., 2017), and errors (Sucec et al., 2018a). Future studies systematically varying the level of dyspnea as well as the level of task difficulty are therefore needed when using the respiratory forced choice reaction time task for the investigation of the impact of unpredictability on interoceptive ERN and task accuracy.

Notably, the present study only observed the effects of unpredictability on anxiety ratings, startle probe N100 and interoceptive ERN when the unpredictable condition was experienced in the first experimental block. We assume that the unpredictability of upcoming dyspnea is particularly strong and anxiogenic, when it occurs in early experimental phases during which participants have not yet made sufficient experiences with the experimental manipulation. In contrast, when the participants first experienced the predictable condition, in which the magnitude of the threat stimulus (i.e., the resistive load) was similar to that in the unpredictable condition, they might experience less unpredictability and subsequent anxiety during the following unpredictable blocks. However, future studies are certainly needed to investigate these effects in larger samples and by systematically varying the longitudinal time course of unpredictable and predictable dyspnea episodes using more than two respective blocks as presented in the present study.

Finally, additional explorative analyses revealed that higher trait-like anxiety sensitivity was associated with higher ratings for dyspnea unpleasantness during both conditions and higher experimental state anxiety ratings only during the unpredictable condition. These findings suggest that individuals with high anxiety sensitivity are more sensitive for dyspnea experiences in general and respond specifically anxious to unpredictable dyspnea. These findings converge with previous studies in which greater anxiety sensitivity and/or trait-like anxiety has been associated with greater reports of dyspnea (Stoeckel et al., 2015; Schroijen et al., 2016; Herzog et al., 2018). For example, Stoeckel et al. (2015) found higher state and trait anxiety to be related to increased self-reports of dyspnea induced by resistive loads. Similarly, higher trait-like anxiety sensitivity has been shown to relate to other anxious responses such as heightened startle responses in anticipation of threat (Melzig et al., 2008; Nelson et al., 2015b; Benke et al., 2018) and a preference for predictable relative to unpredictable CO_2_ administration (Lejuez et al., 2000). Therefore, targeting increased trait anxiety levels by non-pharmacological interventions in patients with cardiopulmonary and mental diseases seems a promising strategy to alleviate their burden of dyspnea (Yohannes and Alexopoulos, 2014; Livermore et al., 2015; von Leupoldt and Janssens, 2016; Herigstad et al., 2017; Spathis et al., 2017; von Leupoldt, 2017; Reijnders et al., 2019).

Despite several notable findings of the present study, these should be interpreted in light of several limitations. First, the number of tested participants as well as presented startle and occlusion probes was relatively small, which may have precluded finding statistically significant effects of unpredictability on anxiety, startle probe N100 and interoceptive ERN for the whole sample independent of order effects. Second, resistive load-induced dyspnea was merely presented for 2 inspirations, resulting in moderate to strong levels of dyspnea unpleasantness and intensity. Longer dyspnea episodes using even stronger resistive load magnitudes, other less flow-dependent qualities of dyspnea (e.g., CO_2_ inhalation, exercise-induced dyspnea) and/or individualized levels of induced dyspnea (i.e., in % of individual maximal inspiratory pressures) might have amplified the effects of unpredictability, which requires future investigations in larger samples. Finally, the majority of tested participants consisted of young and healthy adults without history of dyspnea-related disease with the majority being female, who might respond differently to dyspnea and anxiogenic stimulation than males. This limits the generalizability of the findings. Therefore, future studies are needed to substantiate the present findings in dyspneic patients, who might experience the unpredictability of dyspnea differently.

## Conclusion

Taken together, the present study demonstrated that unpredictability of upcoming dyspnea increases the perception of dyspnea, especially its affective unpleasantness. Moreover, this effect seems related to increased state and trait anxiety and interoceptive error processing, especially when the occurrence of dyspnea is particularly unpredictable such as in early experimental phases. Future studies are required to further substantiate these findings in patients suffering from dyspnea.

## Data Availability

This study does not include any clinical dataset to be shared. The datasets used and/or analyzed during the current study are available from the corresponding author on reasonable request.

## Ethics Statement

This study was carried out in accordance with the recommendations of the Social and Societal Ethics Committee of the University of Leuven with written informed consent from all subjects. All subjects gave written informed consent in accordance with the Declaration of Helsinki. The protocol was approved by the Social and Societal Ethics Committee of KU Leuven (G-2018-02-1123).

## Author Contributions

AvL, OVdB, and YT contributed to the conception and design of the study. AvL and YT contributed to the collection, analysis, and interpretation of the data. AvL and YT wrote the manuscript with critical input from JQ and OVdB. All authors read and approved the final version of the manuscript.

## Conflict of Interest Statement

The authors declare that the research was conducted in the absence of any commercial or financial relationships that could be construed as a potential conflict of interest.
